# Stopping Speed in the Stop-Change Task: Experimental Design Matters!

**DOI:** 10.3389/fpsyg.2019.00279

**Published:** 2019-02-28

**Authors:** Vera Michaela Gordi, Barbara Drueke, Siegfried Gauggel, Stephanie Antons, Rebecca Loevenich, Paul Mols, Maren Boecker

**Affiliations:** ^1^Institute of Medical Psychology and Medical Sociology, University Hospital of RWTH Aachen University, Aachen, Germany; ^2^Department of General Psychology: Cognition and Center for Behavioral Addiction Research, University of Duisburg-Essen, Duisburg, Germany; ^3^Brain Imaging Facility of IZKF Aachen, University Hospital of RWTH Aachen University, Aachen, Germany

**Keywords:** proactive and reactive inhibition, foreknowledge, SSRT, response inhibition, response delay effect, experimental design

## Abstract

Previous research comparing the speed of inhibiting a motor response in no-foreknowledge vs. foreknowledge conditions revealed inconsistent findings. While some studies found stopping to be faster in the no-foreknowledge condition, others reported that it was faster in the foreknowledge condition. One possible explanation for the heterogeneous results might be differences in experimental design between those studies. Given this, we wanted to scrutinize whether it makes any difference if foreknowledge and no-foreknowledge are investigated in a context in which both conditions are presented separated from each other (block design) vs. in a context in which both conditions occur intermingled (event-related design). To address this question a modified stop-change task was used. In Experiment 1 no-foreknowledge and foreknowledge trials were imbedded in a block design, while Experiment 2 made use of an event-related design. We found that inhibition speed as measured with the stop signal reaction time (SSRT) was faster in the foreknowledge as compared to the no-foreknowledge condition of the event-related study, whereas no differences in SSRT between both conditions were revealed in the block design study. Analyses of reaction times to the go stimulus reflect that participants tended to slow down their go responses in both experimental contexts. However, in the foreknowledge condition of the event-related study, this strategic slowing was especially pronounced, a finding we refer to as strategic delay effect (SDE), and significantly correlated with SSRT. In sum our results suggest that inhibition speed is susceptible to strategic bias resulting from differences in experimental setup.

## Introduction

The stop signal paradigm (SSP; Logan et al., 1984) has a long research tradition in investigating the mechanisms underlying the inhibition of motor responses. In the classical SSP participants have to accomplish two different tasks: Whereas the predominant go trials simply require to respond to the go stimulus with one finger of the left or right hand, in stop trials participants need to withhold this already initiated go response whenever the go stimulus is unexpectedly followed by a stop signal. The so-called horse-race model behaviorally describes participants’ performance during stop trials as a race between go- and stop-processes. These two processes are assumed to “run” independently. Whether participants succeed in stopping the response to the go stimulus depends accordingly on the speed of the go response, the speed of the inhibitory process and the delay between the onset of the go-process and that of the stop-process (stimulus onset asynchrony, SOA). The horse-race model allows for the calculation of the in fact covert speed of the inhibition process (stop signal reaction time, SSRT). The SSRT is considered a valid marker of response inhibition and has meanwhile become a standard measure of inhibition.

As the stop signal is presented externally and presumably unexpectedly this type of motor inhibition is mostly referred to as “reactive stopping” (e.g., Verbruggen and Logan, 2008). However, in many everyday-scenarios the inhibition of response tendencies relies on top-down control as we already know ahead that we will have to inhibit a certain motor response. This type of inhibition, often referred to as “proactive inhibition,” is realized according to an individual’s goals. It is generally assumed that “proactive inhibition” is more valid than “reactive inhibition,” as it accounts for many situations in the real world that require the rapid stopping of an action (Aron, 2011) It is important to state at this place that the nomenclature of “reactive stopping” is often used in literature as a description of how stopping is accomplished in the context of the classical SSP. This is unfortunately somewhat misleading, as it has been shown in the last years that even the “simple” classical SSP involves reactive as well as proactive mechanisms (e.g., Zandbelt et al., 2013). We therefore follow the suggestion of Zandbelt et al. (2011) to define reactive inhibition as all reactive mechanisms directly triggered by the stop signal and to define proactive inhibition as all proactive mechanisms that are active when participants expect a stop signal before it is actually presented.

The focus of more recent motor inhibition research has been the question whether foreknowledge about the response that one might have to stop alters performance in inhibition tasks. Therefore, Aron and Verbruggen (2008) introduced an extension of the classical SSP. In contrast to the classical SSP, participants initiate not just one but two simultaneous responses with one finger of each of their hands in response to the presentation of two go stimuli. On a stop trial they are then required to withhold the reaction of one hand, while the other hand still has to execute the go reaction. In order to investigate the influence of foreknowledge on inhibition, participants are either provided with foreknowledge about the response they might need to inhibit (foreknowledge condition), or they do not receive such knowledge (no-foreknowledge condition). This is done by presenting a cue at the beginning of each trial. In the foreknowledge condition the cue is informative and indicates which one of the two hands has to withhold the response in case of a stop trial. In the no-foreknowledge condition the cue is uninformative. This experimental setup not only allows for a comparison of the SSRT (as an indicator of reactive inhibition) in the foreknowledge condition as compared to the no-foreknowledge condition, but in addition the calculation of the amount of interference that stopping one hand produces on the response that still must be conducted by the other hand (stopping interference effect, SIE). Typically, interference is reduced when foreknowledge is given and thus the measure indicates that participants make use of the cue to prepare to stop a specific response tendency (Claffey et al., 2010). From that perspective, the SIE is one way to assess the proactive element in motor inhibition.

Another elegant way to compare proactive inhibition mechanisms between both conditions is to include non-critical vs. critical go trails into the paradigm (e.g., Chikazoe et al., 2009). On non-critical trials a go response is definitely required whereas critical go trials signal that either a go or a stop reaction has to be conducted. Typically, participants delay their responses to the go stimulus when they anticipate a stop signal (critical go trial) as compared to when they do not anticipate a stop signal (e.g., Verbruggen and Logan, 2008, 2009; Zandbelt et al., 2013), which increases the chances of being able to stop in case stopping is required (Logan et al., 1984). Jahfari et al. (2010) referred to this observation as the response delay effect (RDE), which is probably realized by an “active braking mechanism” that is put on the response that one might have to stop without canceling it completely (Jahfari et al., 2010). Previous research reported that a greater RDE was associated with faster stopping (Chikazoe et al., 2009; Jahfari et al., 2010). This observation might indicate that proactive inhibition (reflected by the RDE) makes reactive inhibition (reflected by the SSRT) more efficient.

Based on several studies, Aron et al. (2007) and colleagues proposed a behavioral and neural model of motor inhibition for the no-foreknowledge (Aron and Poldrack, 2006) as well as for the foreknowledge condition (Aron, 2011). Based on this model and the results of a pioneering study comparing both conditions on a pure behavioral level (Aron and Verbruggen, 2008), it was concluded that in a foreknowledge condition stopping is slower (higher SSRT) and interference is smaller (lower SIE). Although there are some studies reporting these exact results (Claffey et al., 2010; Lavallee et al., 2014), other studies found both measures to be lower in the foreknowledge condition (Smittenaar et al., 2013, 2015). Notably, all studies cited varied regarding the experimental design: In the experiments conducted by Claffey et al. (2010) and Lavallee et al. (2014) foreknowledge and no-foreknowledge trials were presented separately in different blocks. In these block design studies the type of inhibition thus remained the same for a number of consecutive trials. Other studies made use of an event-related design, in which foreknowledge and no-foreknowledge trials were randomly presented within the same blocks (Smittenaar et al., 2013, 2015). Interestingly, block design studies report a higher SSRT and a smaller SIE in the foreknowledge condition (Aron and Verbruggen, 2008; Claffey et al., 2010; Lavallee et al., 2014), while event-related design studies found both measures (Smittenaar et al., 2013, 2015) to be lower. From the reported studies comparing inhibition processes in foreknowledge vs. no-foreknowledge conditions, only the event-related design study by Smittenaar et al. (2013) reported results regarding the RDE. However, the authors did not report whether such proactive response slowing was associated with SSRT.

Taken together, previous studies have shown that experimental design might have an effect on reactive (SSRT) as well as proactive inhibitory mechanisms (SIE, RDE). The aim of the present study was therefore to systematically investigate for the first time whether it matters if foreknowledge and no-foreknowledge conditions are compared in a block- vs. event-related the experimental design while holding all other aspects of experimental setup constant across conditions. In two experiments (block design vs. event-related design) we investigated reactive and proactive inhibition processes with the stop change task, an extension of the SSP which will be described in more detail in the method section. We tested the prediction that differences in experimental design impact upon reactive and proactive inhibitory mechanisms. Coupled with this prediction and in accordance with the results of previous studies we generated two hypotheses: First, for the block design study (Experiment 1) we expected SSRT to be higher and SIE to be lower in the foreknowledge condition. Second, for the event-related design study (Experiment 2) we expected both measures to be lower in the foreknowledge condition.

## Experiment 1: Block Design Study

### Methods

#### Participants

Forty right-handed adults (23 women, 17 men, age: *M* = 22.9 ± 2.8 years), mainly students, participated in a 2-h experimental session in return for a monetary reward of €20. Participants reporting psychoactive drug intake, major medical, neurological diseases and/or current mental disorders were excluded from participation. Before the experiment was conducted participants provided written informed consent and completed a health questionnaire. The study protocol was approved by the local Ethics Committee of the Medical Faculty RWTH Aachen University (EK 146/14). All subjects gave written informed consent in accordance with the Declaration of Helsinki.

#### Apparatus

Participants were seated approximately 50 cm in front of a computer with a 23-inch monitor and placed their hands over the keyboard. The experiment was run using Presentation, Version 17.1 ^1^.

#### Materials and Procedure

##### The stop-change paradigm

An extension of the classical SSP, the so called stop-change paradigm (SCP) (Logan et al., 1984) seems to be suited for the examination of foreknowledge and no-foreknowledge conditions. The SCP differs from the classical SSP insofar as it considers that real-life situations require an adaption of an action rather than its complete inhibition. Besides the predominant go trials it therefore contains trials in which participants have to inhibit an already initiated response and subsequently execute an alternative action. Previous research has found only minor differences between SSP and SCP since reaction times (RTs) on go and stop trials are quite similar (for a review see Boecker et al., 2013). Importantly, SSRT, which is commonly referred to as change signal reaction time (CSRT) in the SCP, is comparable in both paradigms. Further, both tasks seem to activate the same brain circuits (Boecker et al., 2013) and rely on the same inhibitory process (Band and van Boxtel, 1999; Verbruggen and Logan, 2008). What makes the SCP unique is that the so called change reaction time (CRT) can be derived, which refers to the time interval between presentation of the signal to change and conductance of the change response. We believe this additional index of response re-engagement can provide valuable information on the mechanisms behind stopping with foreknowledge vs. stopping without foreknowledge as differences between both conditions might indicate in how far the change response had been prepared in advance. To the best of our knowledge, the present study is the first implementing the SCP for a comparison of behavioral inhibitory performance between foreknowledge and no-foreknowledge conditions.

##### Trial types of the stop-change paradigm

**FIGURE 1:**
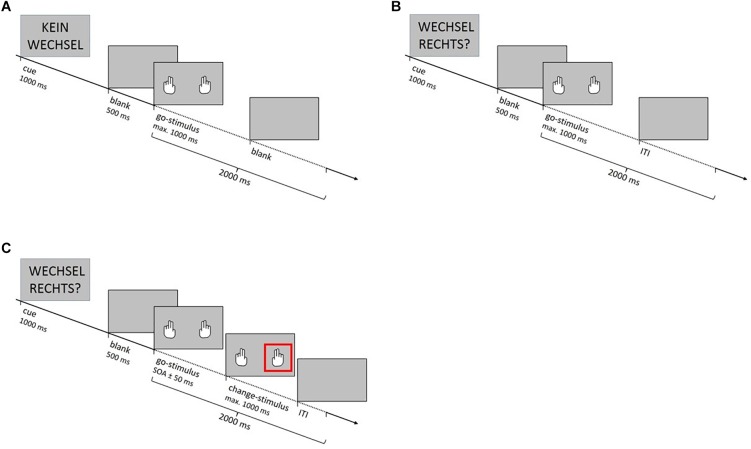
**(A)** On non-critical go trials the cue “No change” (“Kein Wechsel” in German) signaled that a go response was required for certain. **(B)** Cues on critical go trials signaled that either a go or a change reaction was required. In the foreknowledge condition cues were “Change right?” and “Change left?” (“Wechsel rechts?” or “Wechsel links?” in German) and indicated which hand had to execute the alternative response in case of a change trial. In the no-foreknowledge condition the cue “Change xxx?” (“Wechsel xxx?” in German) was uninformative. **(C)**. On change trials the go stimulus was unpredictably followed by the change signal after a variable delay (stimulus onset asynchrony, SOA). The change signal was represented by a red square framing either the right or the left hand. It indicated that participants had to refrain from executing the go reaction with the respective hand and instead press the key assigned to the middle finger (change reaction). When participants gave their response or after 1 s in which no response occurred, the trial terminated with a blank screen (ITI).

The SCP comprised three different trial types (Figure 1). “Non-critical go trials” signaled that a go response was definitely required and “critical go trials” signaled that either a go or a change reaction was required. On both trial types participants had to respond to the go stimuli (pictures of white hands simultaneously presented on a background in light gray) as quickly as possible by pressing the keys assigned to their left and right index fingers once the two hands appeared (go reactions). On “change trials” one of the go stimuli was unpredictably followed by the change signal. The change signal was represented by a red square framing either the right or the left hand and indicated that participants had to refrain from executing the go reaction with the respective hand and instead press the key assigned to the middle finger (change reaction), while for the other hand the go reaction was still required. The change signal appeared after a variable delay (SOA). For calculating the SOA, a tracking algorithm targeting a changing accuracy of 50 percent across all participants was adopted (Kaernbach, 1991). In each condition, the SOA between go stimuli and change signal was initially set at 250 ms for each of the four fingers. After a change trial, it was adjusted for the respective finger according to the individual’s performance: If the stimulus participant successfully executed the change response, the SOA increased by 50 ms, making successful changing on the next change trial more difficult. If the participant failed to execute the change response, the SOA was reduced by 50 ms, making successful changing more likely. Written and verbal instructions underlined that fast and accurate responses were equally important and that delaying the response to the go stimulus in anticipation of the change signal should be avoided. Participants were additionally informed that due to the tracking procedure they would fail to inhibit the already initiated go response in about 50 percent of all change trials.

All go and change trials started with a cue. In the foreknowledge condition cues were “Change right?” or “Change left?” and indicated which hand had to execute the alternative response in case of a change trial. In the no-foreknowledge condition the cue “Change xxx?” was uninformative, as it only indicated that a change signal might be presented. However, it did not indicate which hand might need to execute the alternative response. The trial type “No change!” signaled that the participant was dealing with a go trial (non-critical go trial). When participants gave their response or after 1 s in which no response occurred, the trial terminated with a blank screen (ITI).

##### Distribution of the trial types across conditions

Table 1 demonstrates that there were 100 non-critical go trials. They were equally distributed between foreknowledge and no-foreknowledge condition. Further, each condition consisted of 324 critical go trials and 160 change trials. Thus, the total number of trials included in the experiment was 1068 (70 percent go trials, 30 percent change trials). The conditions were presented in a block design and the order of the starting-condition was counterbalanced across participants. Each condition was split into two equally sized blocks. Change trials were equally distributed between left and right hand. During three breaks after each 267-trial-block participants filled out a demographic questionnaire and the “Edinburgh Handedness Inventory” (Oldfield, 1971). Before participants started with the actual experiment, they performed two practice blocks to get familiar with the task: a block of 18 trials containing only go trials was followed by a block of 53 trials, of which 30 percent were change trials. Participants received a visual feedback “Not simultaneously pressed” whenever responses for the two hands differed by more than 70 ms to undermine a strategy of slowing down the response of the cued hand.

**Table 1:** Experimental conditions and dependent variables.

	Trial type	Cue	N of trials	Dependent variable	Description
	Non-critical go	“No change!”	100	RT (ms)	RT of go trials of which participants know that no change signal will be displayed (averaged across both hands)
**Foreknowledge condition**	Critical go	“Change right?” “Change left?”	324	Cued-hand RT (ms)	RT of the hand that possibly had to change, when the trial turned out to be a go trial
				Non-cued hand RT (ms)	RT of the hand that had to conduct the go response in case of a change trial, when the trial turned out to be a go trial
				RDE	RT between critical and non-critical go trials
				Errors (%)	Percentage of errors on go trials
	Change	“Change right?” “Change left?”	160	SOA (ms)	Delay between appearance of go and change stimuli
				Failed Change RT (ms)	RT of the hand that failed to conduct the change reaction in trials requiring one
				CRT (ms)	Interval between presentation of the change signal and conductance of the accurate alternative response
				Alternative-hand RT (ms)	RT for the hand having to perform a go reaction on change trials
				*p* (change)	Percentage of successful change trials out of all change trials
				SSRT	Stop signal reaction time
				SIE	Stopping interference effect
**No-Foreknowledge condition**	Critical go	“Change xxx?”	324	Non-cued-hand RT (ms)	RT of the hand that had to conduct the go response in case of a change trial, when the trial turned out to be a go trial
				Errors (%)	Percentage of errors on go trials
				RDE	RT between critical and non-critical go trials
	Change	“Change xxx?”	160	SOA (ms)	Delay between appearance of go and change stimuli
				Failed Change RT (ms)	RT of the hand that failed to conduct the change reaction in trials requiring one
				CRT (ms)	Interval between presentation of the change signal and conductance of the accurate alternative response
				Alternative-hand RT (ms)	RT for the hand having to perform a go reaction on change trials
				SSRT	Stop signal reaction time
				SIE	Stopping interference effect
				*p*(change)	Percentage of successful change trials out of all change trials


*RT, reaction time; RDE, response delay effect; SOA, stimulus onset asynchrony; CRT, change reaction time; SSRT, change signal reaction time; SIE, stopping interference effect.*

#### Data Analysis

Two participants incorrectly responded with a change reaction on all trials in the foreknowledge condition and were were therefore excluded. Another participant achieved an extremely small number of successful change trials (<20 percent), leaving 37 participants for the analyses of the participants’ individual RTs. Individuals’ RTs were excluded if one or more of the following criteria applied: (1) RTs were faster than 100 ms, (2) responses of the hands were decoupled by more than 70 ms, and (3) RTs that deviated from the individual mean by more than two standard deviations. After correcting the individual’s data, group means were calculated. Three subjects were excluded from further analyses due to go RTs deviating more than two standard deviations from the group mean. Another subject was excluded because go RTs between the conditions deviated by more than 300 ms, leaving 33 participants for the final analyses.

Dependent variables (see Table 1 for an overview) were computed as follows: First, mean RTs for non-critical go trials were calculated. Next, for critical go trials in the foreknowledge condition cued and non-cued hand RTs were estimated (in case the cue was “Change right?” the right hand represented the cued hand and the left hand represented the uncued hand). For critical go trials in the no-foreknowledge condition mean go RTs of right and left hand was calculated.

The inclusion of non-critical go trials in addition to the critical go trials allowed for a calculation of the RDE. The RDE quantifies the restraint with which the go response is conducted when stopping might be required. Past studies using extended versions of the SSP reported that greater response slowing impacts upon the SSRT (Chikazoe et al., 2009; Jahfari et al., 2010). We therefore decided to examine whether this was the case in the SCP. In both conditions RDEs were calculated as the difference in RT between critical go responses and non-critical go responses.

Stopping speed is usually referred to as CSRT in the stop change paradigm and as SSRT in the classical SSP. In order to keep things simple and due to the fact that the indices SSRT and CSRT have been used interchangeably in metaanalytic reviews, we refer to the speed of stopping as SSRT and not CSRT in the present study. It was calculated by integrating the go RT distribution (Oosterlaan et al., 1998; Lijffijt et al., 2005): First, RTs of the go RT distributions were rank-ordered. In a subsequent step the distribution’s RT representing the probability of failing to change [*p*(signal/unsuccessfulChange)] was selected. Next, the average SOA was subtracted from the RT at *p*(signal/ unsuccessfulChange).

Next, the independence of go and change processes was tested by inspecting the go-RTs and failed change-RTs for each participant as recommended by Logan et al. (1984). For both conditions, go-RTs of the cued hand and failed change-RTs were compared statistically with paired *t*-tests. Additionally, Pearson’s product moment correlations between go-RTs and SSRTs were calculated.

To estimate the amount of interference that stopping one hand produces on the response that still must be conducted by the other hand (SIE), go RTs of the continuing hand were rank-ordered. Then, the median RT of the go RTs exceeding *p*(signal/ unsuccessfulChange) was calculated and subtracted from the median RT of the continuing hand on all change trials (Majid et al., 2013). As a third index, the CRT was represented by the interval between presentation of the change signal and conductance of the accurate alternative response. For accurate change trials, all CRTs were averaged. For successful change trials, the mean go RT for the hand having to perform a go reaction on change trials was assessed (referred to as the “alternative-hand RT”).

Next, *p*(change) was calculated as the percentage of successful change trials out of all change trials.

In a further step we calculated the percentage of errors on critical go trials consisting of omission errors (percentage of go trials on which a response was omitted) and decoupling errors (percentage of go trials on which the hands were decoupled by more than 70 ms).

When foreknowledge and no-foreknowledge condition were compared regarding SSRT and SIE, the results of a recent study pointed to a negative correlation between both measures. The phenomenon was referred to as “speed-selectivity trade-off” (Smittenaar et al., 2013) and indicates that participants either deteriorated in speed and improved in interference or vice versa. At the same time the results of the study demonstrated that most participants benefited from foreknowledge in both measures, SSRT and SIE. Following the example of Smittenaar et al. (2013) the speed-selectivity trade-off was estimated as follows: We first calculated (1) the difference in SSRT between no-foreknowledge and foreknowledge condition and (2) the difference in SIE between no-foreknowledge and foreknowledge conditions. In a second step Pearson’s product moment correlation between the two measures was calculated.

For all key measures, group mean values were analyzed with repeated measures analyses of variance (ANOVA) with condition as within subject factor [non-critical go RT, cued-hand RT, non-cued hand RT, RDE, SSRT, SIE, CRT, alternative-hand RT, *p*(change)] using SPSS (IBM SPSS 22, 2013). Additionally, effect sizes were calculated using Cohen’s *d*. Pearson’s product moment correlations between SSRT and RDE were calculated to reveal whether participants who delay more would be able to stop their responses more quickly.

### Results

For critical go trials (trials with a cue indicating that either a go or a change response would be required) and non-critical go trials (trials that definitely required a go response) paired *t*-tests did not reveal significant differences between RTs of right and left hands (all *p* > 0.24). RTs across right and left hands were therefore pooled for both foreknowledge and no-foreknowledge conditions.

Consistent with the horse-race model, failed change RT was shorter than go RT of the cued hand in both conditions [foreknowledge condition: *t*(32) = -2.30, *p* = 0.03; no-foreknowledge condition: *t*(32) = -5.20, *p* < 0.001]. Additionally, RTs of the cued hand were not related to SSRT (foreknowledge condition: *r* = 0.11, *p* = 0.54; no-foreknowledge condition: *r* = 0.18, *p* = 0.31).

All means and confidence intervals are reported in Table 2. Multivariate analyses of variance revealed a significant effect for the within-subject factor condition [*F*(8,25) = 7.66, *p* < 0.001]. Univariate analysis revealed no significant differences for the main effect of condition for non-critical go responses [*F*(1,32) = 0.96, *p* = 0.33], critical go responses [cued-hand RT: *F*(1,32) = 0.17, *p* = 0.68; non-cued-hand RT: *F*(1,32) = 0.14, *p* = 0.71] and RDE [*F*(1,32) = 0.02, *p* = 0.9], although participants did in fact delay their responses on critical go trials in both conditions. In addition, we did not reveal a significant difference in SSRT between conditions [*F*(1,32) = 2.10*, p* = 0.16]. However, foreknowledge significantly decreased interference [SIE: *F*(1,32) = 19.54, *p* < 0.001], the change reaction time [CRT: *F*(1,32) = 61.9, *p* < 0.001] and the RT of the alternative hand *F*(1,32) = 7.03, *p* = 0.01). There was no difference in the probability of changing between conditions: *F*(1,32) = 0.57, *p* = 0.46. Moreover, the RDE was not related to SSRT (foreknowledge condition: *r* = -0.1, *p* = 0.58; no-foreknowledge condition: *r* = -0.04, *p* = 0.83).

**Table 2:** Means and 95% confidence intervals for Experiment 1 (*N* = 33).

	Experiment 1 (*N* = 33)		
Measure	Foreknowledge	No foreknowledge	Cohen’s *d*
Non-critical GoRT (ms)	277 [250, 304]	269 [245, 293]	0.10 [–0.38, 0.59]
Cued-hand RT (ms)	499 [458, 540]	485 [446, 524]	0.12 [–0.37, 0.60]
Non-cued hand RT (ms)	498 [455, 541]	485 [446, 524]	0.11 [–0.38, 0.59]
Response delay effect (ms)	221 [168, 274]	216 [179, 253]	0.04 [–0.45, 0.52]
Failed change RT (ms)	427 [391, 461]	419 [381, 458]	0.07 [–0,42, 0,56]
SSRT (ms)	276 [264, 288]	288 [268, 308]	–0.25 [–0.73, 0.24]
CRT (ms)	374 [354, 394]	433 [411, 455]	–0.33 [–0.82, 0.15]
Alternative-hand RT (ms)	577 [530, 624]	626 [575, 677]	–0.33 [–0.82, 0.15]
*p*(change)	45 [43,47]	46 [44,48]	–0.22 [–0.70, 0.27]
Errors on go trials (%)	1.5 [1.02, 1.92]	1.0 [0.71, 1.29]	0.42 [–0.9, 0.07]


*Cued-hand RT = RT of the hand that possibly had to change, when the trial turned out to be a go trial; Non-cued hand RT = RT of the hand that had to conduct the go response in case of a change trial, when the trial turned out to be a go trial; Failed Change RT = RT of the hand that failed to conduct the change reaction in trials requiring one; SSRT = Change Signal Reaction Time; SIE = Change Interference Effect; CRT = Change Reaction time; Alternative-hand RT = RT of the hand that conducted the go response on a change trial; *p*(change) = percentage of change trials conducted correctly.*

**FIGURE 2:**
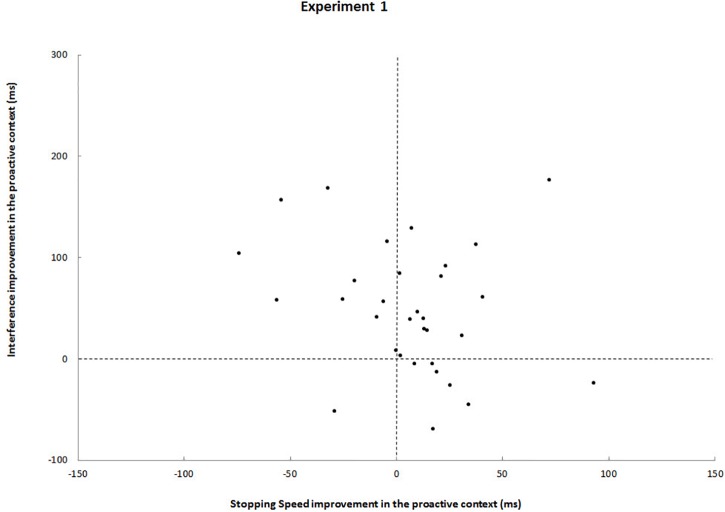
Each black dot represents a participant. Upper left quadrants, participants who improved from foreknowledge in interference but deteriorated in stopping speed; Upper right quadrants, participants who improved from foreknowledge in both interference and stopping speed; Lower left quadrants, participant who deteriorated in both measures; Lower right quadrants, participants who improved from foreknowledge in stopping speed stopping speed but deteriorated in interference.

Further analyses showed no statistically significant speed-selectivity trade-off (*r* = -0.21, *p* = 0.25). Figure 2 underlines that: ten participants improved in interference but deteriorated in stopping speed (upper left quadrant) and seven participants showed the opposite pattern (lower right quadrant). Most participants (*n* = 15), however, even improved from foreknowledge in both interference and stopping speed (upper right quadrant).

### Discussion

Initially, we expected SSRT to be higher and SIE to be lower in the foreknowledge condition. This hypothesis was derived from the results of previous block design studies. In their behavioral study Aron and Verbruggen (2008) found that stopping was faster in the no-foreknowledge condition, a result which could be replicated by Claffey et al. (2010). The authors reasoned that these findings point to an engagement of the indirect pathway during stopping with foreknowledge because the indirect pathway contains a greater number of synaptic connections and therefore transmits the stop signal slower than the hyperdirect pathway (e.g., Mink, 1996), the putative candidate pathway for stopping when foreknowledge is lacking (Aron, 2011). Although our findings demonstrate a lower SIE in the foreknowledge condition, SSRT did not differ between conditions. These contrasting results suggest that transferring behavioral data onto neuronal circuits is not as straightforward as it has been assumed by Aron and Verbruggen (2008). Future imaging studies are required to clarify whether and to what extend activity in inhibitory neuronal circuits differs for foreknowledge vs. no-foreknowledge conditions. Participants delayed their responses to the go-stimuli in critical go trials in both conditions as indicated by the pronounced RDEs. This delay did not differ between conditions. Contrary to previous studies investigating the RDE, the delay did not correlate with the SSRT.

Although the present study made use of a block design similar to the one used by Aron and Verbruggen (2008) we cannot completely rule out that other differences in experimental setup between our study and other block design studies are associated with the deviating findings regarding the SSRT. For example, our study comprised a simple-reaction task, in which the go task was not connected to a discrimination task, whereas in the studies by Aron and Verbruggen (2008) and Claffey et al. (2010) participants conducted a choice-reaction task. They first had to discriminate the location of the go stimulus (e.g., under or above a line/ inside or outside an arrangement of circles) and in a next step conduct the go response with the finger that was specifically assigned to the location of where the go stimulus popped up. The need for cognitive control was thus higher in these choice-reaction tasks since participants had to maintain two goals (press both index fingers when go stimulus shows up above the line vs. press both middle fingers when go stimulus shows up below the line), whereas in our simple reaction tasks there was just one task goal (press both index fingers). However, go RTs between our study and the study by Aron and Verbruggen (2008) are comparable, showing that possible differences in the need for cognitive control are not reflected by higher go RT in choice-reaction tasks.

## Experiment 2: Event-Related Design Study

### Methods

#### Participants

Twenty-six right-handed students (11 women, 15 men, age: *M* = 23.1 ± 2.9 years) who did not participate in Experiment 1 were tested in a 2-h session in return for a monetary reward of €20. Exclusion criteria were the same as in Experiment 1.

#### Apparatus, Materials, and Procedure

Experiment 2 was identical to Experiment 1 except that foreknowledge and no-foreknowledge trials were presented in an event-related design. Number and distribution of go and change trials were the same as in Experiment 1. Before participants started with the experiment, they performed four practice blocks. A block of 11 trials containing only go trials was followed by two blocks of 26 trials, of which 30 percent were change trials. In one of these blocks cues were “Change left?” or “Change right?” while the second block contained the uninformative cue “Change xxx?” only. The last practice block was like the actual experiment and contained 53 trials. The experiment was split into four blocks which were separated from each other by 3-min breaks.

#### Data Analysis

Two participants did not perform the task correctly and were therefore excluded. Another one achieved an extremely small number of successful change trials in the no-foreknowledge condition (<20 percent), leaving 23 participants for the analyses of the participants’ individual RTs. Exclusion criteria were the same as in Experiment 1. After correcting the individual’s data, group means were calculated. Three more subjects were excluded from the analyses due to very slow RTs that deviated more than two standard deviations from the group mean, leaving 20 participants for the main analyses.

All calculations were conducted as in Experiment 1. Due to the fact that trials with and without foreknowledge were presented intermingled, non-critical go trials could not be distributed between the conditions as in Experiment 1.

### Results

Paired *t*-tests did not reveal significant differences between RTs of the right and left hand (all *p* > 0.24). RTs of both hands were therefore pooled.

Consistent with the horse-race model, failed change RT was shorter than go RT of the cued hand in both conditions [foreknowledge condition: *t*(19) = 5.07, *p* < 0.001; no-foreknowledge condition: *t*(19) = 4.86, *p* < 0.001]. Additionally, go RTs of the cued hand were not related to SSRT {foreknowledge condition: *r* = -0.15, *p* = 0.52; no-foreknowledge condition: *r* = -0.06, *p* = 0.8}.

All means and confidence intervals are reported in Table 3. Multivariate analyses of variance revealed a significant effect for the within-subject factor condition [*F*(7,13) = 29.35, *p* < 0.001]. Univariate analysis revealed significant differences for the main effect of condition for critical go responses: On critical go trials reactions to the go stimulus where conducted significantly slower in the foreknowledge condition [cued-hand RT: *F*(1,19) = 10.12, *p* = 0.005; non-cued-hand RT: *F*(1,19) = 9.87, *p* = 0.005]. Again, comparisons between critical and non-critical were go trials reflect that participants delayed their responses when they were possibly dealing with a trial requiring a change response (see RDEs) and this time the RDE was more profound in the foreknowledge condition [*F*(1,19) = 10.0, *p* = 0.005]. Furthermore, participants improved from foreknowledge in both inhibition measures, interference and stopping [SSRT: *F*(1,19) = 27.49, *p* < 0.001; SIE: *F*(1,19) = 46.59, *p* < 0.001] and the change reaction time were significantly lower when they had foreknowledge [CRT: *F*(1,19) = 160.43, *p* < 0.001]. There were no significant differences for the main effect of condition for the alternative-hand RT [*F*(1,19) = 160.43, *p* < 0.001]. However, participants changed significantly more correctly on change trials in the foreknowledge condition [*F*(1,19 = 14.49, *p* = 0.001].

**Table 3:** Means and confidence intervals for Experiment 2 (N = 20).

	Experiment 2 (*N* = 20)		
Measure	Foreknowledge	No foreknowledge	Cohen’s *d*
Non-critical GoRT (ms)	255 [230, 279]	255 [230, 279]	0.00 [–0.62, 0.62]
Cued-hand RT (ms)	506 [456, 557]	457 [415, 499]	0.49 [–0.14, 1.12]
Non-cued hand RT (ms)	507 [456, 558]	457 [415, 499]	0.49 [–0.14, 1.12]
Response delay effect (ms)	252 [209, 295]	202 [165, 239]	0.72 [0.08, 1.36]
Failed Change RT (ms)	457 [413, 500]	398 [351, 446]	0.56 [–0.1, 1.19]
SSRT (ms)	248 [218, 278]	304 [283, 325]	–0.98 [–1.64, –0.33]
CRT (ms)	337 [322, 352]	441 [418, 464]	–2.46 [–3.27, –1.63]
Alternative-hand RT (ms)	583 [517, 648]	608 [548, 668]	–0.18 [–0.80, 0.44]
*p*(change)	48 [46, 49]	45 [43, 47]	0.74 [0.10, 1.38]
Errors on go trials (%)	1.9 [1.41, 2.39]	1.1 [0.67, 1.61]	0.69 [0.01, 1.14]


*Cued-hand RT = RT of the hand that possibly had to change, when the trial turned out to be a go trial; Non-cued hand RT = RT of the hand that had to conduct the go response in case of a change trial, when the trial turned out to be a go trial; Failed Change RT = RT of the hand that failed to conduct the change reaction in trials requiring one; SSRT = Change Signal Reaction Time; SIE = Change Interference Effect; CRT = Change Reaction time; Alternative-hand RT = RT of the hand that conducted the go response on a change trial; *p*(change) = percentage of change trials conducted correctly.*

It was not the RDE in the foreknowledge condition that was related to SSRT (*r* = -0.08, *p* = 0.73). Instead, we found that the difference in critical go RTs between foreknowledge and no-foreknowledge condition significantly correlated with SSRT in the foreknowledge condition (*r* = -0.6, *p* = 0.01). Thus, greater differences in go RTs between the conditions were directly associated with a faster inhibition process in the foreknowledge condition. We named this observation “strategic delay effect” (SDE).

**FIGURE 3:**
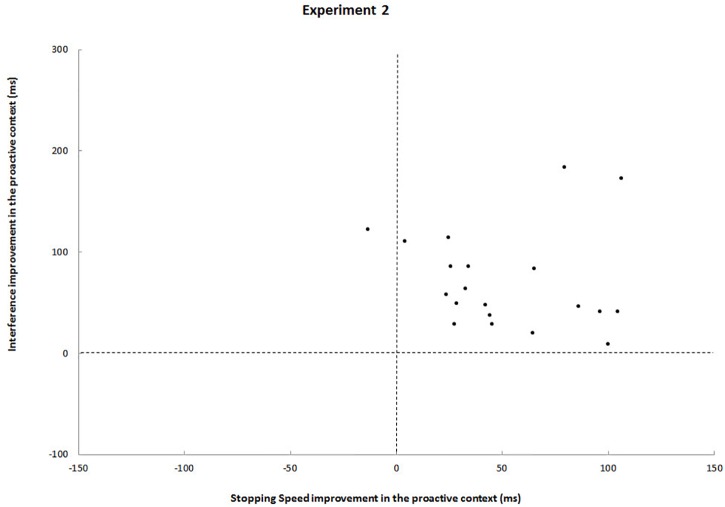
Each black dot represents a participant. Upper left quadrants, participants who improved from foreknowledge in interference but deteriorated in stopping speed; Upper right quadrants, participants who improved from foreknowledge in both interference and stopping speed; Lower left quadrants, participant who deteriorated in both measures; Lower right quadrants, participants who improved from foreknowledge in stopping speed stopping speed but deteriorated in interference.

Again, a statistically significant speed-selectivity trade-off could not be revealed (*r* = 0.20, *p* = 0.4). On the contrary, a comparison between block and event-related design shows that the event-related design drastically reduced data heterogeneity: when foreknowledge was available, 19 out of 20 participants improved in both stopping speed and interference (Figure 3).

### Discussion

In line with our hypothesis and the results of previous event-related design studies (Smittenaar et al., 2013, 2015) we found SSRT and SIE to be lower in the foreknowledge condition. RTs (Table 3) show that inhibition was more efficient when foreknowledge was provided. This result is in agreement with the findings of another event-related study (Smittenaar et al., 2013). At the individual level, however, the results of Smittenaar et al. (2013) are more heterogeneous than ours. Although the authors found that for most participants SSRT and SIE were lower in the foreknowledge condition, there was one group who benefited in speed only and another one who benefited in interference only, a finding they refer to as speed-selectivity-trade-off. In contrast, such a trade-off could not be revealed in our event-related study, as for 19 out of 20 participants inhibition in both measures was more efficient when foreknowledge was provided (Figure 3). On top of that we found that the RDE differed between conditions in our event-related study, which again contrasts the results of the study by Smittenaar et al. (2013).

Such differences between studies suggest that experimental design is just one among several other experimental factors that have an impact on inhibition measures such as SSRT and SIE. Although the study design was the same, there were other experimental factors such as the cue-stimulus interval, the amount of trials (both inhibit and go) and stimulus material that differed from the study by Smittenaar et al. (2013). Future studies may reveal the differential contribution of these components to the heterogeneous results reported here.

## General Discussion

The present article aimed to clarify whether experimental design impacts upon inhibition speed in an extended version of the stop change paradigm. We demonstrate that this is indeed the case: In the block design study (Experiment 1) SSRT did not differ between foreknowledge and no-foreknowledge conditions. In the event-related study (Experiment 2), however, SSRT was lower in the foreknowledge condition as compared to the no-foreknowledge condition.

Another striking difference between both designs is reflected by effect sizes: Although interference (SIE) and CRT were reduced in the foreknowledge condition of the block design as compared to the no-foreknowledge condition, these effects were much greater in the event-related design. Apart from that, no difference between critical go responses and RDE was observed between conditions of the block design study; whereas large effects were found in the event-related design, with greater critical go RTs and greater RDEs in the foreknowledge condition. We refer to the latter observation as the SDE. The negative correlation between SDE and SSRT underlines that proactive inhibition might contribute to make reactive inhibition more efficient. Moreover, data heterogeneity was substantially smaller in the event-related design, reflecting that this experimental setup seemed to reinforce the use of a more uniform response strategy across participants (Figure 2 vs. Figure 3). In the following sections we will take a closer look at the possible strategies used by the participants and the way these strategies differed between conditions and designs.

In both our experiments substantial RDEs were found. Importantly, this was the case for both foreknowledge and no-foreknowledge conditions, reflecting that the simple anticipation of a stop signal leads to an activation of proactive mechanisms (Logan et al., 1984; Vink et al., 2014). The fact that participants delay their responses to the go signal when they anticipate a stop signal (RDE) applies not only for the classical SSP (e.g., Logan, 1981; Logan and Burkell, 1986) but also for its extensions such as the conditional stop task (Jahfari et al., 2010) or the stop signal anticipation task (Zandbelt et al., 2011; Vink et al., 2014). Although in the present work such strategic adjustments of the go response were shown for both designs and both conditions, there was one striking difference: Contrasting the results of our block-design study, critical go RTs in the event-related study show that participants slowed down more in the foreknowledge compared to the no-foreknowledge condition. It thus seemed that once foreknowledge about which response participants might have to change was provided, the event-related design led to the strategy of even further delaying the response to the go signal, whereas this was not the case in the block-design study (SDE). We assume that the SDE reflects the creation of two motor plans in the foreknowledge condition (press both index fingers versus press one index finger and the other hand’s middle finger) and just one motor-plan in the no-foreknowledge condition (press both index fingers). The preparation of two motor-plans would probably be more time-consuming and therefore explains the higher go RTs foreknowledge condition. The preparation of two motor-plans also serves as an explanation for the observed decreases in interference and CRT, as the availability of the second motor-plan possibly allowed for a quicker initiation of the alternative-response as well as the change response.

Why didn’t we find a SDE in the block design? We believe that direct comparison between foreknowledge and no-foreknowledge trials is necessary for participants to adopt different strategies for responding to these two conditions. Due to the time lag between the block that contains only no-foreknowledge trials and the block containing only foreknowledge trials, direct comparison is rather impossible in a block design. However, the lack of difference in go RT does not imply that participants refrain from responding strategically. A block design reduces cognitive load because there are only two cues in the foreknowledge condition (“Change left?” or “Change right?”) and just one cue in the no-foreknowledge condition (“Change xxx?”). What do participants use the extra cognitive capacities for then? We believe it is possible that adjustments in response thresholds are made throughout the course of one block of trials, maybe even on a trial-by-trial base as has been suggested previously (Verbruggen and Logan, 2009; Smittenaar et al., 2013). A block design would thus promote the switching between different response thresholds, which would also explain the greater data heterogeneity observed in the block design study. Future studies are needed to examine possible trial-by-trial adaptations to the go stimulus when participants are faced with a block design.

As stated above, it is well documented that once participants expect a stop signal to occur, go RT increases, an effect that is even larger when the objective stop signal probability as well as the subjective expectation of a signal to occur increases (Vink et al., 2015). Most probable, such proactive response slowing is the outcome of an increased response threshold (Verbruggen and Logan, 2009) for the initiation of the go response in the primary cortex (Jahfari et al., 2010). Thus, during response slowing the critical response is partially suppressed, although not completely canceled (Jahfari et al., 2010). Functional imaging studies have repeatedly shown that brain regions active during anticipation of stopping (proactive inhibition) and reactive inhibition overlap (Chikazoe et al., 2009; Jahfari et al., 2010; Zandbelt et al., 2011), with separate activation in left and right striatum, the supplementary motor complex (SMC), and the midbrain during presentation of the cue (Zandbelt et al., 2013). Especially activity in the striatum increases as stopping becomes more probable (Zandbelt and Vink, 2010).

Originally, it has been proposed that SSRT remains unaffected from proactive response slowing (Logan et al., 1984). However, the finding that in Experiment 2 the SDE (as a measure of proactive inhibition) was directly associated with the SSRT (as a measure of reactive inhibition) speaks against this. On the contrary, our result leads to the suggestion that proactive and reactive inhibitory mechanisms not only interact on a neuronal but also on a behavioral level. Other studies showing that proactive adjustments lead to differences in SSRT seem to confirm this assumption. For example, via rewards and penalties and the manipulation of task instructions Leotti and Wager (2010) targeted either responses toward correct stopping or responses toward fast going. Their results indicate that targeting correct stopping lead to decreases in SSRT and increases in go RT while the reverse applied when fast going was targeted. Complementing the results of Leotti and Wager (2010), other researchers found that the strategy of response slowing decreases SSRT (Chikazoe et al., 2009; Jahfari et al., 2010). In another study SSRT systematically varied within subjects as a function of stop signal probability, although the effect was small (Jahfari et al., 2012). These findings and the fact that in the present study strategically delaying go responses lead to faster stopping suggest that the SSRT may depend on participants’ proactive response strategies. It is quite understandable that the validity of SSRT as a measure of inhibition processing and the horse-race model, on which the SSRT is based on, have been doubted in the context of these results. The question arises whether the extended versions of the SSP are sufficiently similar to the classical SSP to allow for transferring the assumptions of the horse-race model. Another problem is that complete independence between go- and stop-processes is unlikely, as both processes are known to interact in certain neural networks (Hanes et al., 1998) and might therefore better be reflected by an interactive race model (Boucher et al., 2007). Nevertheless, we show that in our experiments the assumption of the independent race model is met, which is in line with previous studies comparing inhibition processes in foreknowledge and no-foreknowledge conditions (Smittenaar et al., 2013, 2015). In any case, there is an urgent need to again consider the validity of the independent race model to further understand the paradox between participants’ strategic response slowing in stop signal tasks and its influence on SSRT on the one hand, and the proposed independence of go- and stop-processes on the other hand.

There is a limitation of our study that needs to be considered. We noted that experimental differences between studies impede comparability between studies. Nevertheless, the present study made use of a modified stop-change task, which to date has not been used for comparing inhibition with and without foreknowledge. Our study thus does not actually contribute to an easier interpretation of inhibition measures. However, we decided on conducting our research with the SCP as (1) the task considers that real-life situations require an adaption of behavior rather than its complete inhibition and (2) provides us with the CRT, an extra index of response inhibition processes which other extensions of the SSP do not provide for. As a valuable extra information, the CRT revealed that participant’s preparation for conducting the change response was also more efficient when foreknowledge was available, an effect that was even greater in the event-related design.

Taken together, the present research provided first evidence that inhibition as measured by SSRT, SIE and CRT when foreknowledge is available is more effective in an event-related design. Furthermore, we could show that in the event-related design, greater proactive inhibition makes reactive inhibition more efficient “when foreknowledge is available”. As to these results, we believe our study could serve as a first stepping stone for further investigating proactive and reactive inhibition processes in the condition of differences in experimental setup.

## Data Availability

The raw data supporting the conclusions of this manuscript will be made available by the authors, without undue reservation, to any qualified researcher.

## Author Contributions

VG, MB, BD, and SG participated in the conception and design of the study. PM programmed the experiment and prepared analysis routines. VG, SA, and RL prepared the study material and collected the data. VG performed the statistical analysis. VG, MB, BD, and SG contributed substantially to interpreting the data. VG drafted the manuscript. All authors contributed to manuscript revision, read and approved the submitted version.

## Conflict of Interest Statement

The authors declare that the research was conducted in the absence of any commercial or financial relationships that could be construed as a potential conflict of interest.

## Footnotes

1 www.neurobs.com
